# Myocardial KChIP2 Expression in Guinea Pig Resolves an Expanded Electrophysiologic Role

**DOI:** 10.1371/journal.pone.0146561

**Published:** 2016-01-14

**Authors:** Drew M. Nassal, Xiaoping Wan, Haiyan Liu, Isabelle Deschênes

**Affiliations:** 1 Heart and Vascular Research Center, Department of Medicine, MetroHealth Campus, Case Western Reserve University, Cleveland, Ohio, United States of America; 2 Department of Physiology and Biophysics, Case Western Reserve University, Cleveland, Ohio, United States of America; University of South Florida, UNITED STATES

## Abstract

Cardiac ion channels and their respective accessory subunits are critical in maintaining proper electrical activity of the heart. Studies have indicated that the K^+^ channel interacting protein 2 (KChIP2), originally identified as an auxiliary subunit for the channel Kv4, a component of the transient outward K^+^ channel (*I*_*to*_), is a Ca^2+^ binding protein whose regulatory function does not appear restricted to Kv4 modulation. Indeed, the guinea pig myocardium does not express Kv4, yet we show that it still maintains expression of KChIP2, suggesting roles for KChIP2 beyond this canonical auxiliary interaction with Kv4 to modulate *I*_*to*_. In this study, we capitalize on the guinea pig as a system for investigating how KChIP2 influences the cardiac action potential, independent of effects otherwise attributed to *I*_*to*_, given the endogenous absence of the current in this species. By performing whole cell patch clamp recordings on isolated adult guinea pig myocytes, we observe that knock down of KChIP2 significantly prolongs the cardiac action potential. This prolongation was not attributed to compromised repolarizing currents, as *I*_*Kr*_ and *I*_*Ks*_ were unchanged, but was the result of enhanced L-type Ca^2+^ current due to an increase in Cav1.2 protein. In addition, cells with reduced KChIP2 also displayed lowered *I*_*Na*_ from reduced Nav1.5 protein. Historically, rodent models have been used to investigate the role of KChIP2, where dramatic changes to the primary repolarizing current *I*_*to*_ may mask more subtle effects of KChIP2. Evaluation in the guinea pig where *I*_*to*_ is absent, has unveiled additional functions for KChIP2 beyond its canonical regulation of *I*_*to*_, which defines KChIP2 as a master regulator of cardiac repolarization and depolarization.

## Introduction

K+ channel interacting proteins (KChIPs) represent a class of highly diverse Ca^2+^-sensors originally discovered for their interaction with the cytoplasmic N-terminus of the Kv4 family of potassium channels [1]. The assembly of these two proteins creates the native current known as A-type current (*I*_*A*_) in neuronal tissue and the fast-inactivating transient-outward potassium current (*I*_*to*,*f*_*)* in the heart [2–6]. While the expression of Kv4 alone is sufficient to observe this current, co-expression with KChIP results in currents with slowed inactivation, faster recovery from inactivation, and increased current densities, effectively creating native Kv4 current [1, 7].

In total, there are four KChIP genes (KChIP1-4) [1, 8]. While all four KChIPs can be observed in the brain, the heart is seemingly simplified by expressing only KChIP2. Together, these proteins are characterized by a highly conserved C-terminal domain containing 4 EF-hand motifs and a highly variable N-terminus which is thought to provide both altered localization and activity [9, 10]. Indeed, the KChIP family has become the most diverse of the Ca^2+^-sensing proteins [11, 12] not just in numbers, but in their breadth of function. In addition to serving as K^+^-channel subunits, KChIP3 and 4 were discovered in the brain to both regulate presenilin [8, 13], affecting the processing of amyloid precursor protein. Additionally, KChIP3 was discovered to act as a transcriptional repressor, an activity later shown by all four KChIP isoforms [14, 15]. Even cardiac KChIP2 expression shows behavior of interacting with more than Kv4, including Kv1.5, which has been shown to display impaired trafficking by KChIP2 [16]. Additionally, there is evidence that KChIP2 interacts with the N-terminus of Cav1.2, affecting channel open probability [17, 18], while we have shown a regulation on *I*_*Na*_ through a potential macromolecular interaction with *I*_*to*_ [19].

This concept of multimodal function is reinforced in the setting of the guinea pig where we show expression of myocardial KChIP2 is maintained despite having a complete absence of Kv4 expression [20], making it unclear what function KChIP2 satisfies. Frequently, mice have been used to investigate the impact of KChIP2 expression, where KChIP2 loss invariably diminishes *I*_*to*_, the main repolarizing current in rodents, and therefore prolongs the cardiac action potential. However, the guinea pig offers a unique perspective to investigate what other influences emerge as a consequence of KChIP2 without the dominant influence of *I*_*to*_ present. Here, we demonstrate using the guinea pig that KChIP2 possesses additional functions beyond its canonical regulation of *I*_*to*_ that define KChIP2 as a master regulator of cardiac repolarization and depolarization.

## Materials and Methods

### Detection of relative kcnip2 (KChIP2) expression across species

Left ventricular tissue was excised from the hearts of rat, guinea pig, dog, and human samples. Both ventricles were taken from neonatal rat hearts. Tissue was immediately frozen in liquid nitrogen and pulverized on dry ice for better homogenization. Total RNA was isolated from the pulverized heart tissue using Trizol Reagent (Invitrogen) according to the manufacturer’s instructions. 20 ng/μl RNA per sample was used as a template for cDNA synthesis in reverse transcriptase reactions using the Multiscribe Reverse Transcriptase kit (Invitrogen). Real-time quantitative PCR reactions were performed with the ABI 7500 Real-Time PCR system using Power SYBR green PCR Master Mix (Invitrogen) technology. Relative quantification of *kcnip2* across samples was normalized using *gapdh* and the 2^∆∆Ct^ method was implemented to determine relative quantification. A melting curve was performed to verify amplification of discrete products. All primer pairs used spanned at least a single intron to select against any contaminating genomic amplification. The following primers were used for detection: *rat kcnip2*.*F*: 5’-ACTTTGTGGCTGGTTTGTCG-3’, *rat kcnip2*.*R*: 5’- TGATACAGCCGTCCTTGTTGAG-3’; *rat gapdh*.*F*: 5’- AGTTCAACGGCACAGTCAAG-3’, *rat gapdh*.*R*: 5’- ACTCCACGACATACTCAGCAC-3’; *guinea pig kcnip2*.*F*: 5’- AGAAACAAGGATGGCGTGGT-3’, *guinea pig kcnip2*.*R*: 5’- CAAAGAGCTGCATGGATCGC-3’; *guinea pig gapdh*.*F*: 5’- GCGCCGAGTATGTAGTGGAA-3’, *guinea pig gapdh*.*R*: 5’- TGATTCACGCCCATCACGAA-3’; *canine kcnip2*.*F*: 5’- ACGTATCCTGCACTCCGAGA-3’, *canine kcnip2*.*R*: 5’- GCCATCCTTGTTCCTGTCCA-3’; *canine gapdh*.*F*: 5’- GGGCGTGAACCATGAGAAGT-3’, *canine gapdh*.*R*: 5’- CAGTGATGGCATGGACGGT-3’; *human kcnip2*.*F*: 5’- TGTACCGGGGCTTCAAGAAC-3’, *human kcnip2*.*R*: 5’-GGCATTGAAGAGAAAAGTGGCA-3’, *human gapdh*.*F*: 5’- TCCTCTGACTTCAACAGCGA-3’, *human gapdh*.*R*: 5’- GGGTCTTACTCCTTGGAGGC-3’

### Viral Contructs

cDNA of the identified guinea pig KChIP2 antisense sequence was created and cloned into an adenoviral vector under the regulation of a CMV promoter (KChIP2-KD). The vector coexpressed GFP through an internal ribosomal entry site. A control adenoviral vector was used that omitted the antisense sequence and expressed GFP alone.

### Immunoblotting

Freshly isolated guinea pig ventricular myocytes were cultured for 24 h at 37°C in M199 medium under control conditions or with KChIP2 antisense virus. Upon collection, cardiomyocytes were washed in ice-cold PBS and resuspended in a RIPA lysis buffer (150 mM sodium chloride, 1.0% NP-40 or Triton X-100, 0.5% sodium deoxycholate, 0.1% SDS (sodium dodecyl sulphate), 50 mM Tris, pH 8.0, plus Roche Inhibitor tablet) and then sonicated on ice to disrupt cell membranes. 30–40 μg of whole cell extract was loaded into SDS-PAGE gels, transferred to nitrocellulose membrane, and Western blotting performed using KChIP2 mouse monoclonal antibody (UC Davis NeuroMab 75–017) at a 1:500 dilution, beta-actin mouse monoclonal antibody (Sigma-Aldrich, A1978) at a 1:1000 dilution, Cav1.2 mouse monoclonal antibody (UC Davis NeuroMab 75–257) at a 1:500 dilution, Nav1.5 mouse monoclonal antibody (Sigma S8809) at a 1:800 dilution, and pan-cadherin rabbit monoclonal antibody (Cell Signaling 4068S) at a 1:1000 dilution. Protein concentrations were determined by the BCA method (Pierce).

### Guinea Pig Ventricular Myocytes: Isolation and Short-term Culture

Single ventricular myocytes were isolated from adult guinea pigs as described previously [21]. Briefly, guinea pigs were anesthetized by injection of fatal plus. Hearts were quickly removed and perfused via the aorta with a physiological salt solution (PSS) containing (in mmol/L) NaCl 140, KCl 5.4, MgCl_2_ 2.5, CaCl_2_ 1.5, glucose 11, and HEPES 5.5 (pH 7.4). After 5 minutes, perfusate was switched to a nominally calcium-free PSS with collagenase (Roche, 0.5 mg/mL) being added after an additional 5 minutes. After 20 to 35 minutes of digestion, hearts were perfused with a high K^+^ solution containing (in mmol/L) potassium glutamate 110, KH_2_PO_4_ 10, KCl 25, MgSO_4_ 2, taurine 20, creatine 5, EGTA 0.5, glucose 20, and HEPES 5 (pH 7.4). Ventricles were minced in high K^+^ solution, and single myocytes were obtained by filtering through a 115-μm nylon mesh. Myocytes were left to settle for 2 hours at room temperature before being collected in a low-speed spin. Cell pellets were resuspended in M199 medium supplemented with antibiotic and plated on uncoated 10 cm dishes. Cultures were left untreated or they were treated with GFP adenovirus or adenovirus with a *kcnip2* (KChIP2) mRNA antisense coding sequence in IRES with GFP. Cultures were then incubated in 5%CO_2_ at 37°C for 24 hrs. Untreated and GFP treated cells were found to have no significant differences across the studies conducted, and therefore the datasets between these two groups were combined to comprise our control group.

### Cellular Electrophysiology

*I*_K1,_ *I*_*Ks*_, *I*_*Kr*_, and action potentials were recorded in isolated ventricular guinea pig myocytes cultured overnight in M199 medium using the following intracellular solution: 119 mM potassium gluconate, 15 mM KCl, 3.75 mM MgCl_2_, 5 mM EGTA, 5 mM HEPES, 4 mM K-ATP, 14 mM phosphocreatine, 0.3 mM Tris-GTP, and 50 U/ml creatine phosphokinase, pH 7.2. The extracellular solution was 132 mM NaCl, 4.5 mM KCl, 1.2 mM MgCl_2_, 1.8 mM CaCl_2_, 10 mM Glucose, and 5 mM HEPES, pH 7.4. *I*_*K1*_ currents were elicited from a holding potential of -40 mV with depolarizing voltage pulses from -100 mV to 40 mV. *I*_*Ks*_ currents were elicited from a holding potential of -40 mV with depolarizing voltage pulses from -30 mV to 60 mV for 2.5 s and then return to -40 mV to generate outward tail currents in the presence of 5 uM E4031 to block *I*_*Kr*_. *I*_*Kr*_ currents were isolated as E4031 sensitive current. *I*_*Ca*,*L*_ were recorded with an intracellular solution of 130 mM CsMES, 20 mM TEA Cl, 1 mM MgCl_2_, 10 mM HEPES, 10 mM EGTA, 0.3 mM TRIS GTP, 14 mM Phosphocreatine, 4 mM Mg ATP, 2 mM Creatine phosphokinase and brought to a pH of 7.3. Myocytes were placed in the solution containing 137 mM NaCl, 5.4 mM CsCl, 1.8 mM MgCl_2_, 2 mM CaCl_2_, 10 mM glucose, 10 mM HEPES, pH 7.3. *I*_*Ca*,*L*_ were elicited from a holding potential of -40 mV with depolarizing voltage pulses from -30 mV to 60 mV for 300 ms. *I*_*Na*_ was recorded in the solution containing 20 mM NaCl, 120 mM N-methyl D-glucamine, 5.4 mM CsCl, 1 mM MgCl_2_, 10 mM glucose, 10 mM HEPES, pH 7.3. 1 uM of nisodipine was used to block L-type Ca currents. *I*_*Na*_ were elicited from a holding potential of -120 mV with depolarizing voltage pulses from -80 mV to 60 mV for 16 ms. Ionic current density (pA/pF) was calculated from the ratio of current amplitude to cell capacitance. pClamp software (Molecular Devices) was used for generation of voltage-clamp protocols and data acquisition. All experiments were performed at 35°C except *I*_*Na*_ (room temperature).

### Statistical Analysis

The experimental data were expressed as mean ± SEM. A Student’s *t*-test or a paired Student’s *t*-test (for Western blot analysis) was performed. All tests were two-sided and a significance level of p < 0.05 was defined as statistically significant (SPSS 18.0 software, SPSS, Chicago, IL)

### Ethics Statement and Tissue Acquisition

This study was carried out in strict accordance with the recommendations in the Guide for the Care and Use of Laboratory Animals of the National Institutes of Health. The protocol for tissue isolation from adult guinea pig (Protocol Number: 2012–0175), adult and neonatal rat (Protocol Number: 2013–0015), and canine (Protocol Number: 2014–0026) samples were approved by the Committee on the Ethics of Animal Experiments of Case Western Reserve University. 14 Hartley guinea pigs, 3 adult Sprague Dawley rats, and 3 neonatal hearts from 3 separate litters were provided from Charles River to conduct our studies. 3 separate hearts of canine left ventricular tissue were sourced from purpose bred canines from Marshall Farms. Tissue from the left ventricular free wall of 3 non-failing, human samples were acquired from the Cleveland Clinic Foundation (CCF) tissue repository from unmatched organ donors. All protocols were approved by the CCF Institutional Review Board (IRB# 2378). Samples were received coded and no identifying metrics were documented for the study.

## Results

**Fig 1:**
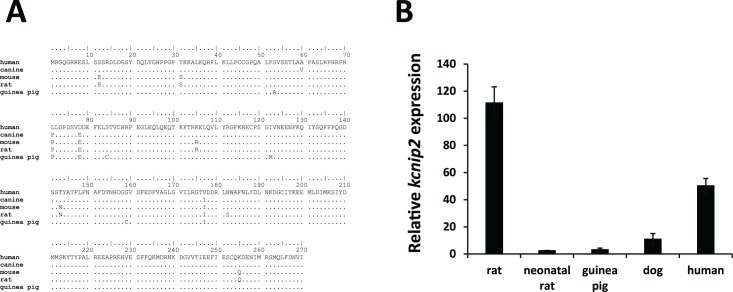
Conservation and expression of KChIP2 across species. **(A)** Protein sequence alignment across multiple mammals shows a high degree of sequence homology for KChIP2. **(B)** Relative expression of *kcnip2* (KChIP2) mRNA isolated from ventricular tissue (presented as means ± SEM, n = 3, guinea pig n = 5). Guinea pig expression is significantly less than other species but comparable to neonatal rat tissue where *I*_*to*_ is present.

We began our investigation by first identifying the maintained expression of KChIP2 in the guinea pig myocardium. Sequence alignment of the identified transcript shows significant sequence homology compared to other mammalian species which possess Kv4 encoded *I*_*to*_ (Fig 1A)_._ Variability exists, however, in the relative levels of *kcnip2* (KChIP2) mRNA expression. Left ventricular tissue isolated from guinea pig expresses approximately 35-fold less KChIP2 than the ventricular tissue from an adult rat (Fig 1B), making the level of KChIP2 mRNA expression comparable to neonatal rat ventricular myocytes. Previous work has established the degree of KChIP2 expression directly correlates with the amount of *I*_*to*_ density [22–24]. Indeed, adult rat which has high levels of KChIP2 has the most prominent *I*_*to*_ density of the animals shown, while neonatal rats which express much less KChIP2 has a smaller *I*_*to*_ density which reflects the lower expression [25]. An important distinction, however, is that neonatal rat myocytes display measurable *I*_*to*_, while the guinea pig myocardium, while maintaining comparable KChIP2 levels expresses no *I*_*to*_, suggesting a functional consequence of KChIP2 presence beyond the modulation of *I*_*to*_.

**Fig 2:**
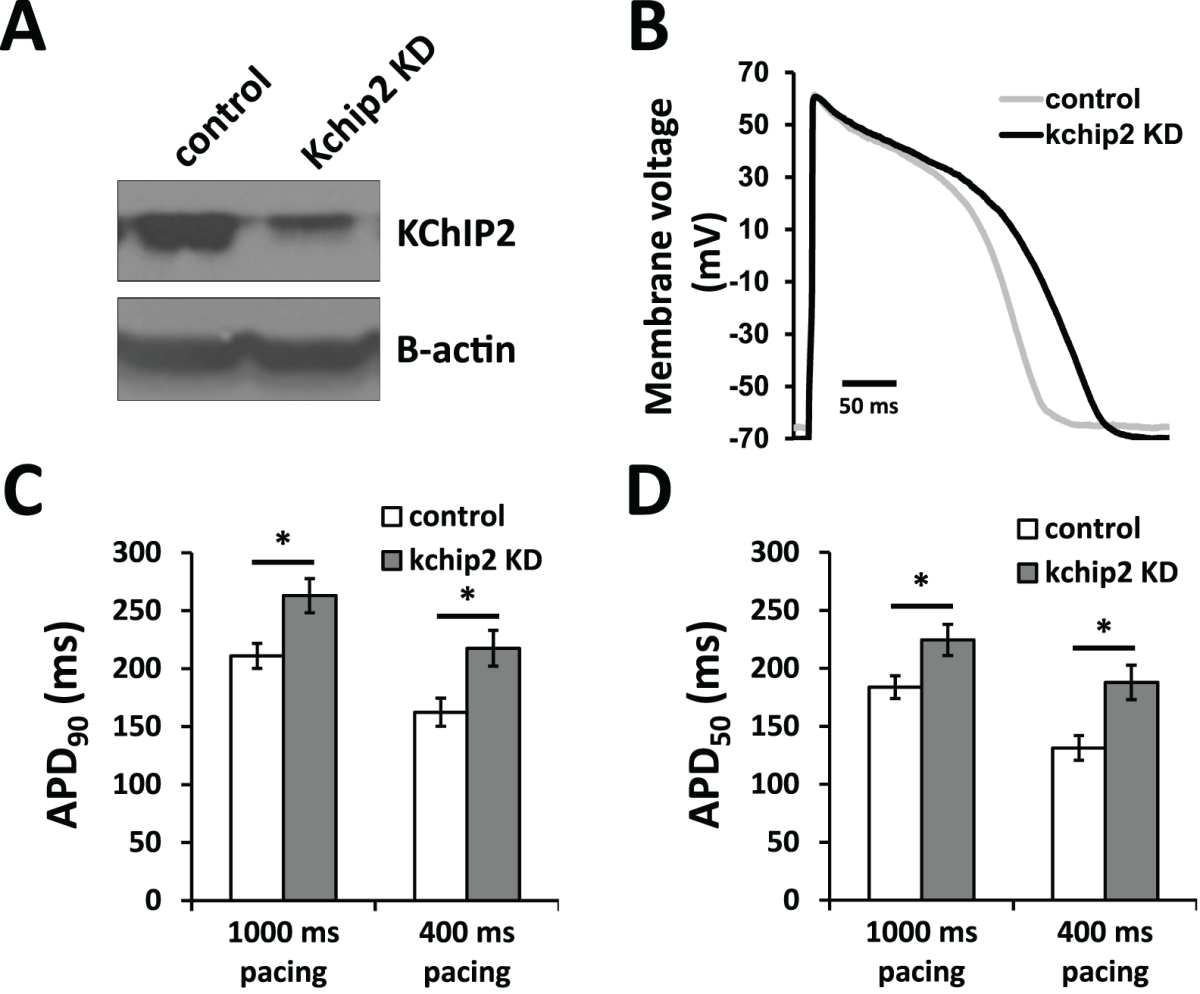
KChIP2 knockdown in isolated ventricular myocytes of the guinea pig prolongs the cardiac action potential. **(A)** Immunoblot for KChIP2 of whole cell lysate isolated from ventricular cells treated for 24 hrs with adenovirus encoding either GFP (control) or an mRNA antisense sequence for KChIP2 (KChIP2 KD). Beta-actin was used as a loading control **(B)** Representative action potential tracings at 1000 ms cycle length from isolated ventricular cells following 24 hrs incubation with Ad.KChIP2-KD. **(C)** Summary data of APD_90_ during 1000 ms and 400 ms cycle lengths. KChIP2 KD cells at 1000 ms (n = 16) show a significant prolongation of APD, compared to control cells (n = 14). KChIP2 KD at 400 ms (n = 11) was also significantly prolonged, compared to control cells (n = 9). **(D)** APD_50_ following 1000 ms and 400 ms cycle lengths for the same treatment groups. Data presented as mean ± SEM; *P < 0.05; two-tailed Student’s *t*-test.

**Fig 3:**
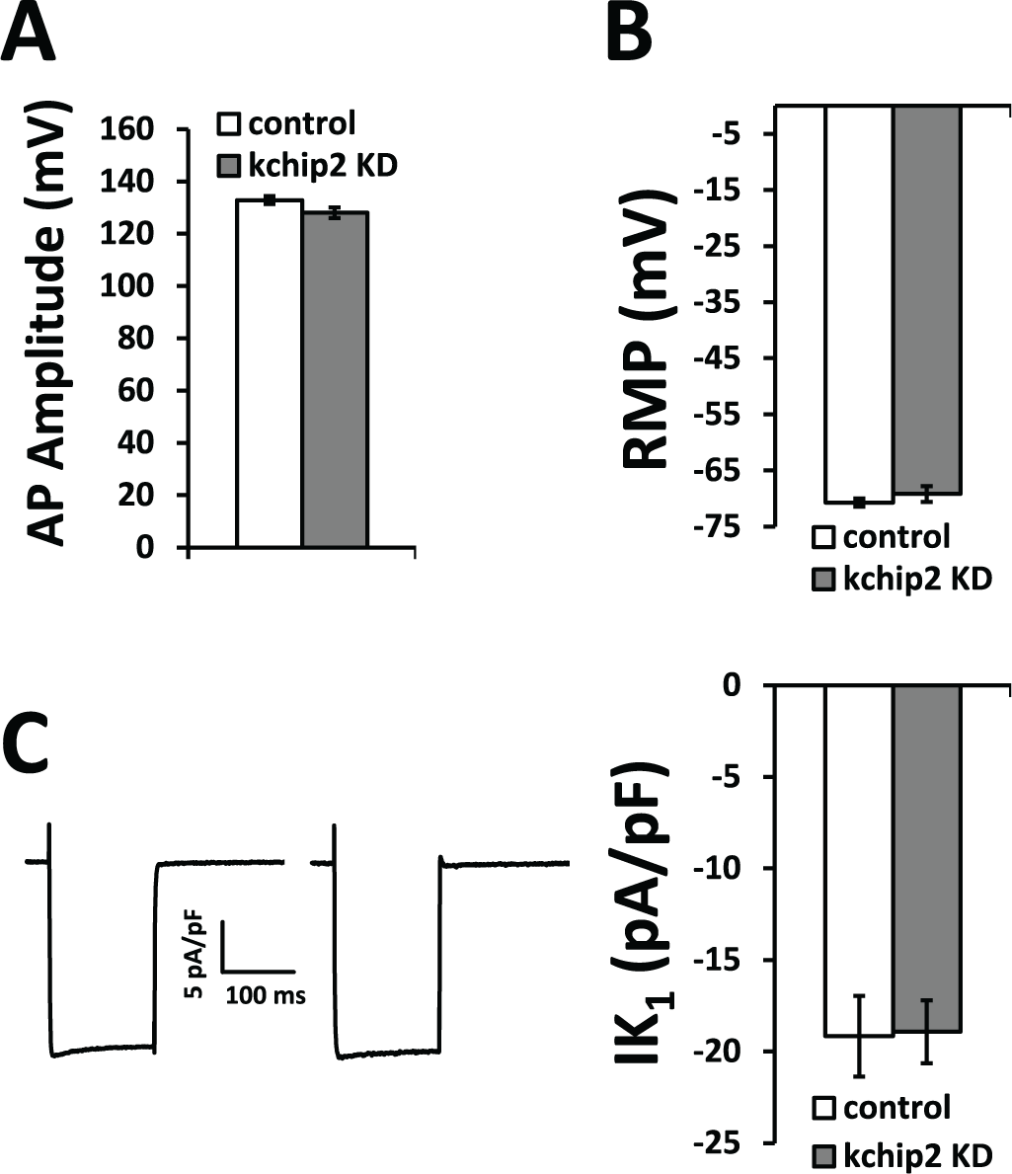
Action potential parameters. **(A)** Action potential amplitude and **(B)** resting membrane potential between control (n = 14) and Ad.KChIP2-KD (n = 16) isolated cardiomyocytes at 24 hrs shows no significant difference. **(C)** Left panel shows representative peak *I*_*K1*_ elicited by a voltage step to -100 mV from a holding potential of -40 mV. Right panel shows summary data of these peak averages, indicating no significant change between control (n = 11) and KChIP2 KD (n = 10) cardiomyocytes. Data presented as mean ± SEM; two-tailed Student’s *t*-test performed.

To begin to address if guinea pig cardiac KChIP2 contributes to cardiac excitability and modulation of other ionic currents, we evaluated the guinea pig action potential following acute changes to KChIP2 expression. Ventricular cells isolated from the adult guinea pig heart were left untreated or were treated for 24 hrs with an adenovirus expressing GFP or an mRNA antisense sequence for KChIP2 to acutely silence KChIP2. Evaluation by Western blot shows we were able to significantly reduce KChIP2 protein in KChIP2 antisense treated cells compared to control cells (Fig 2A). Critically, the consequence of this reduction was significant prolongation of the cardiac action potential, extending APD_90_ from 211±10.8 ms in control cells to 262±14.6 ms in anti-sense treated cells (Fig 2B). This prolongation was significant at both 90% and 50% of repolarization and occurred at multiple pacing frequencies (Fig 2C and 2D). The overlap at phase 1 of the action potential traces between both groups is reflective of the absence of *I*_*to*_ or any other comparable repolarizing current that KChIP2 has been shown to effect. This data implicates a clear influence of KChIP2 on factors beyond *I*_*to*_. Notably, action potential amplitude, resting membrane potential, and *I*_*K1*_ density were all unaltered by reductions in KChIP2 (Fig 3A, 3B and 3C), showing that these factors were not responsible for changing action potential morphology or duration.

**Fig 4:**
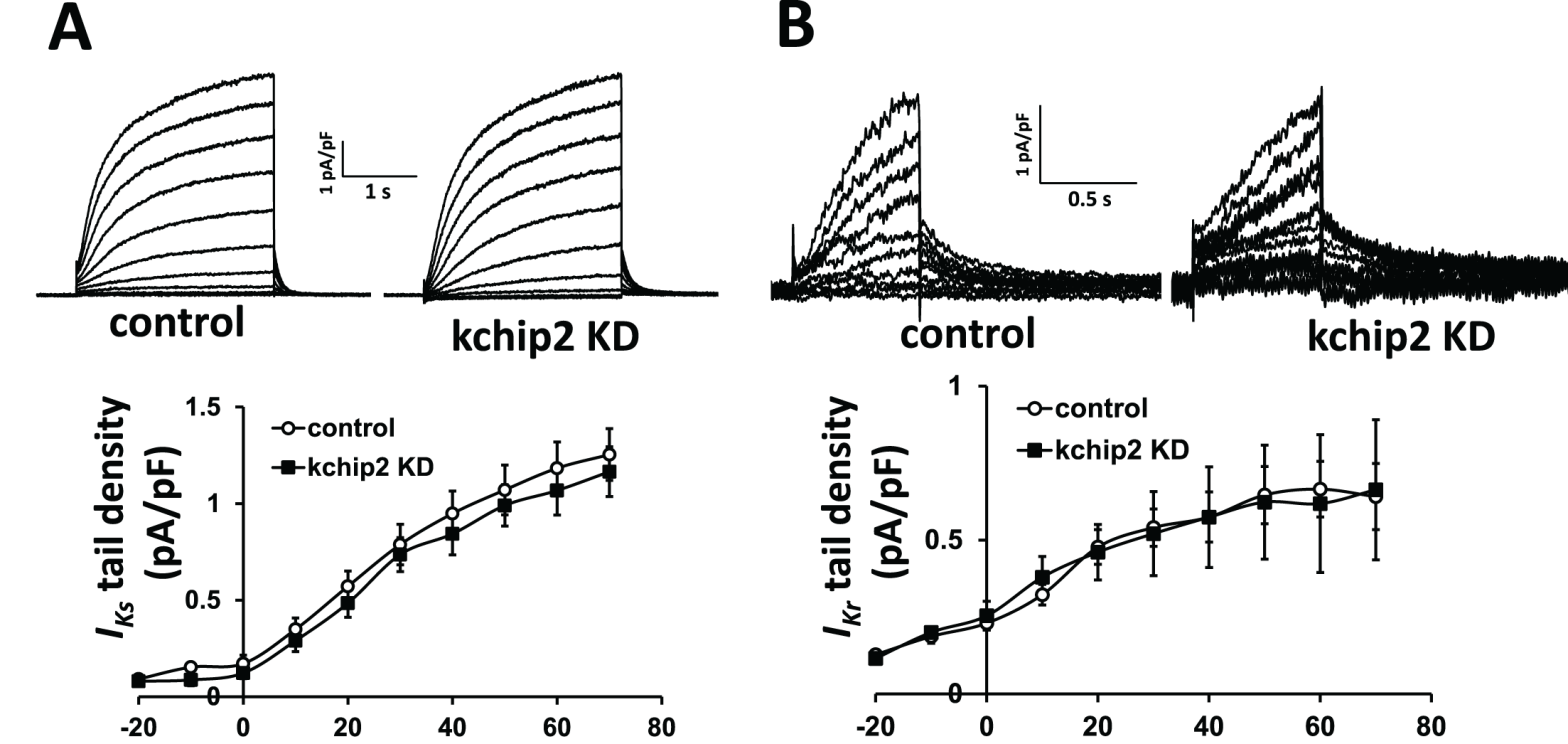
APD prolongation observed following KChIP2 silencing is not defined by compromised repolarization. **(A)** Upper panel shows representative current traces for *I*_*Ks*_ for both control and KChIP2 KD cardiomyocytes following 24 hrs incubation. Currents were generated from a holding potential of -40 mV with depolarizing voltage pulses from -30 mV to 60 mV, and then a return to -40 mV to generate outward tail currents in the presence of E4031. Lower panel shows the resulting I/V curve summary data between control (n = 19) and KChIP2 KD (n = 10) yielding no significant difference between treatment groups. **(B)** Upper panel shows representative current traces for *I*_*Kr*_ for both control and KChIP2 KD cardiomyocytes following 24 hrs incubation. Currents were isolated as E4031 sensitive. Lower panel shows the resulting I/V curve summary data for tail currents between control (n = 9) and KChIP2 KD (n = 4). There was no significant difference between the two groups. Data presented as mean ± SEM; two-tailed Student’s *t*-test performed.

**Fig 5:**
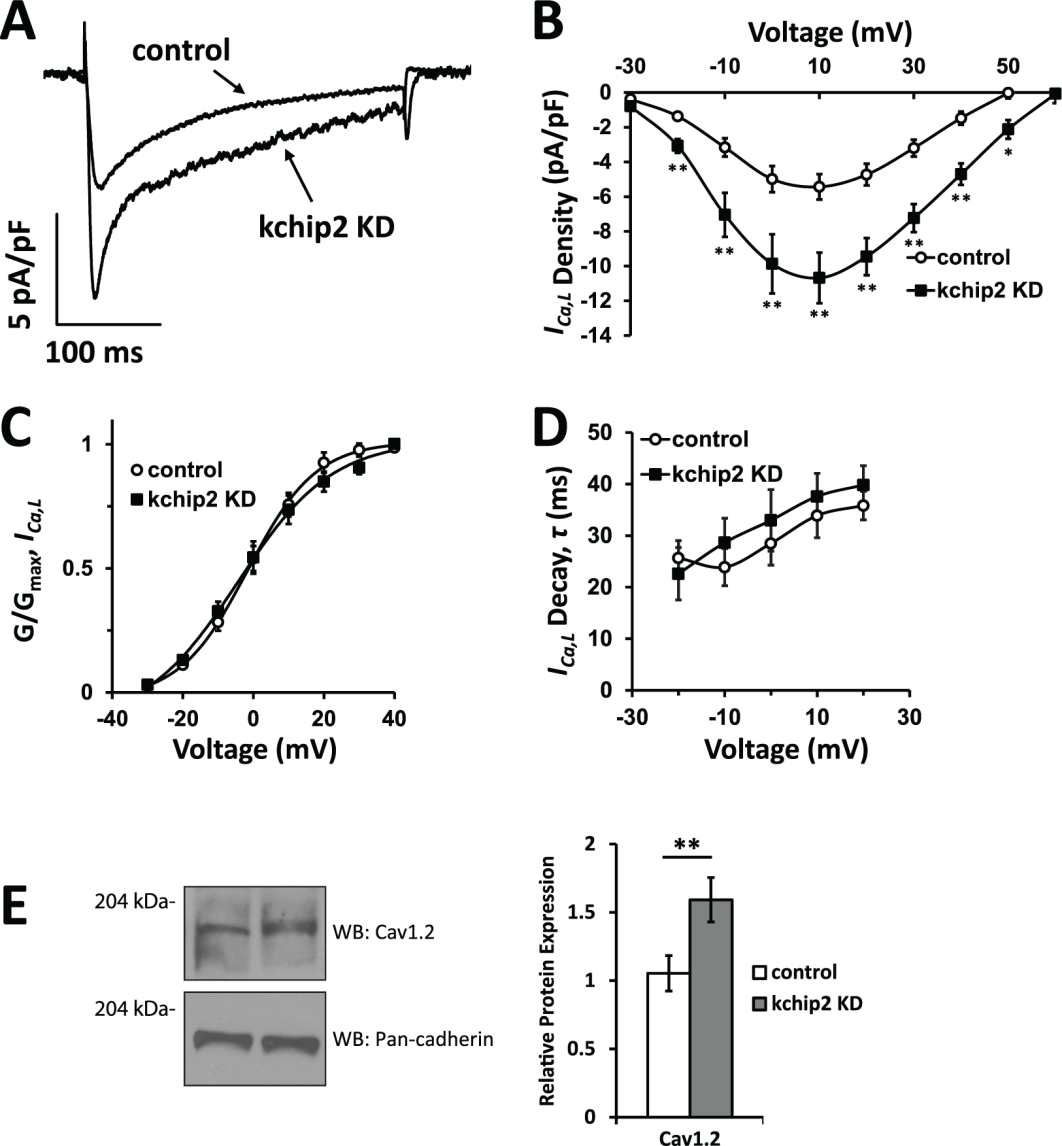
KChIP2 knock down enhances density of L-type Ca2+ current. **(A)** Representative *I*_*Ca*,*L*_ current traces of control and KChIP2 KD treated cells following 24 hrs incubation. Currents were elicited from a holding potential of -40 mV and a depolarizing step to 10 mV. **(B)** I/V curve summary data of *I*_*Ca*,*L*_ displays significant enhancement of current density following KChIP2 KD (n = 12) compared to control cardiomyocytes (n = 20), explaining the prolonged plateau phase of the cardiac action potential. **(C)** Steady-state activation of *I*_*Ca*,*L*_ in control and KChIP2 KD treated myocytes. The data, depicted as normalized conductance, was fit with a Boltzmann curve but no significant difference was detected at any test pulse. **(D)** Evaluation of current decay kinetics between control and KChIP2 KD treated myocytes at several membrane voltages. *τ*-values were derived from single exponential fits of *I*_*Ca*,*L*_ decay following channel activation, also showing no significant difference at any test pulses. **(E)** Left panel shows representative immunoblots for Cav1.2 protein from whole cell lysates for control and KChIP2 KD treated myocytes. Protein expression was normalized to pan-cadherin expression. Right panel shows summary data depicting the normalized, relative densitometry of Cav1.2 expression, resulting in significantly increased expression following KChIP2 KD (n = 4). Data presented as mean ± SEM; *P < 0.05, **P < 0.01; two-tailed Student’s *t*-test performed for I/V plot, paired two-tailed Student’s *t*-test performed for Western blot.

We next sought to identify the currents responsible for the prolonged repolarization observed following KChIP2 silencing. Given the observed prolongation of the plateau phase of the guinea pig action potential, we focused on changes to *I*_*Kr*_, *I*_*Ks*_, and *I*_*Ca*,*L*_. While there is no history for KChIP2 regulation on either of the delayed rectifier currents, we measured their activity to determine if these currents might be responsible for the delayed repolarization. However, assessment of the tail current densities for *I*_*Kr*_ and *I*_*Ks*_ displayed no significant change to either current (Fig 4A and 4B). Overall, the loss of KChIP2 does not appear to affect outward repolarizing currents in the guinea pig myocardium. Therefore, we next looked at potential changes to *I*_*Ca*,*L*_, to determine if enhancement of this depolarizing current might explain a longer APD. Indeed, when looking at the changes to L-type Ca^2+^ current we see that reduction of KChIP2 produced an increase in current density across multiple potentials when compared to control cells (Fig 5A and 5B). Peak current density at 10 mV was enhanced from -5.43±0.73 pA/pF in control cells to -10.68±1.46 pA/pF in KChIP2 anti-sense treated cells, nearly doubling the current density. Analysis of channel activation and voltage-dependent inactivation revealed that the kinetics of *I*_*Ca*,*L*_ were unaltered by KChIP2 KD and could not account for the increase in current density (Fig 5C and 5D). However, assessment of Cav1.2 protein expression showed that KChIP2 KD led to a significant increase in expression compared to control cells (1.85 ± 0.05 fold change, Fig 5E). Given that current kinetics were left unaltered, this suggests *I*_*Ca*,*L*_ augmentation occurs through an increase in channel expression following the loss of KChIP2.

**Fig 6:**
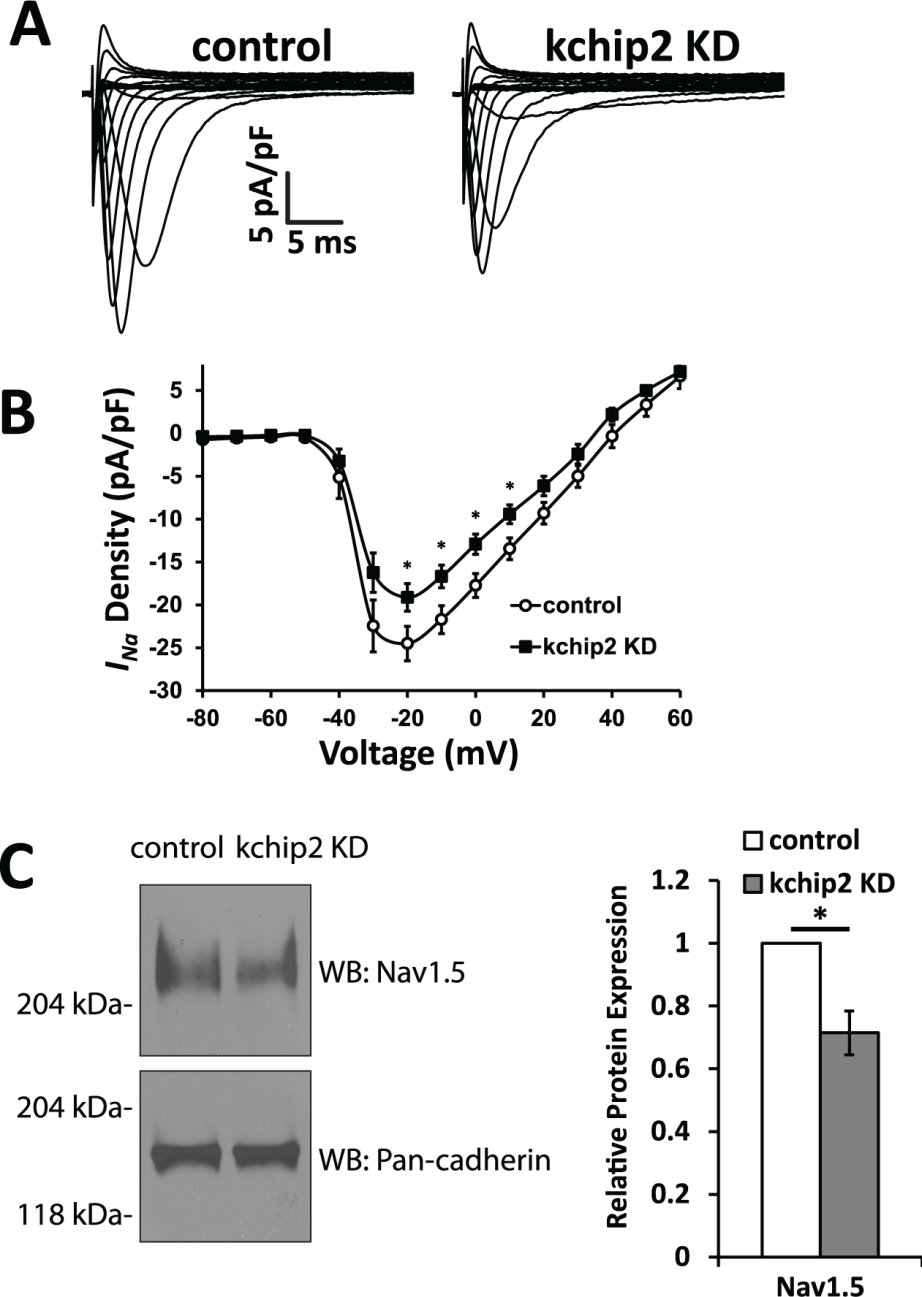
KChIP2 knock down attenuates *I*_*Na*_ density. **(A)** Representative traces for *I*_*Na*_ in cardiomyocytes in control and KChIP2 KD cells following 24 hrs incubation. **(B)** Summary data of the I/V curve for control (n = 13) and KChIP2 KD (n = 12) cardiomyocytes, showing reduced current density in response to KChIP2 loss. **(C)** Left panel shows representative immunoblots for Nav1.5 protein from whole cell lysates for control and KChIP2 KD treated myocytes. Protein expression was normalized to pan-cadherin expression. Right panel shows the average fold change from KChIP2 KD treated cardiomyocytes from control, which resulted in significantly decreased expression following KChIP2 KD (n = 3). Data presented as mean ± SEM; *P < 0.05 two-tailed Student’s *t*-test performed for I/V plot, paired two-tailed Student’s *t*-test performed for Western blot.

Lastly, to evaluate the full panel of currents relevant in the guinea pig cardiac action potential, changes to *I*_*Na*_ was assessed. Evaluation of *I*_*Na*_ density showed a decrease in the peak current from -24.51 ± 1.70 pA/pF in control cells, to -19.14 ± 2.09 pA/pF in KChIP2 knock down treated cells (Fig 6A and 6B). To understand this loss in current, Nav1.5 protein was assessed, which showed a mild but significant decrease following KChIP2 loss (0.71 ± 0.07 fold change, Fig 6C), reflecting the change in current density. This suggests that KChIP2 not only has influence over the rate of repolarization, but may potentially have regulation over cardiac excitability as well.

## Discussion

This study sought to characterize the influence of KChIP2 expression in the guinea pig myocardium. The species was specifically chosen for its unique, endogenous absence of *I*_to_ [20] despite our observation of maintained KChIP2 expression, suggesting an alternative significance for this protein. Additionally, this model provided a means of evaluating the electrophysiological consequences of KChIP2 loss without the influence of diminished *I*_*to*_ density. Indeed, when we silenced KChIP2, we observed a significantly longer plateau phase of the action potential, resulting in a prolonged APD. This prolongation was due to enhanced L-type Ca^2+^ current density, with no change to the repolarizing currents *I*_*Kr*_ and *I*_*Ks*_. Additionally, we measured significant reduction to *I*_*Na*_ density. Together, these data show that KChIP2 is indeed a multimodal effector of cardiac ionic currents.

In rodents, *I*_*to*_ serves as the primary repolarizing current maintaining a strong influence over APD [26]. In response to cardiac stressors like myocardial infarction and pressure overload, both KChIP2 and Kv4 experience reduced expression, significantly contributing to APD prolongation [27–29]. It was found that maintaining KChIP2 expression even in the presence of cardiac stress could normalize the APD back towards baseline levels [30]. This had the therapeutic effect of mitigating the hypertrophic response by minimizing increases in the Ca^2+^ transient and turning off Ca^2+^ activated hypertrophic signaling pathways. In larger mammals, however, this relationship is less straightforward as *I*_*to*_ is comparatively smaller and therefore does not fully repolarize the membrane [31], which means decreased *I*_*to*_ does not necessarily translate to prolonged APD. In fact, data suggests reductions in *I*_*to*_ can actually lead to APD shortening by altering the driving force for Ca^2+^ entry [31][32]. Yet, independent of species, cardiac pathologies almost invariably result in diminished KChIP2 expression and a prolonged APD [33–35]. Therefore, the observations in the guinea pig of KChIP2 loss directly augmenting *I*_*Ca*,*L*_ through increases in Cav1.2 protein (Fig 5) is highly significant in fully understanding its contributions to disease pathogenesis. It shows that independent of its effect on *I*_*to*_, KChIP2 depletion can still prolong the cardiac action potential (Fig 2) and possibly drive disease progression through Ca^2+^ dependent signaling cascades.

This is not the first instance showing that KChIP2 is capable of causing changes to L-type Ca^2+^ current. Thomsen *et al* previously identified in a mouse KChIP2 knockout model a significantly reduced *I*_*Ca*,*L*_ in the absence of KChIP2 [17], the opposite regulation measured in the guinea pig here. This regulation was discovered to be the consequence of KChIP2 interaction with an N-terminal auto-inhibitory (NTI) domain on Cav1.2, which was interrupted when KChIP2 was bound, resulting in an increased open probability for the channel. However, when identifying this NTI domain in the guinea pig, alignment with the mouse shows an incomplete conservation of the amino acid residues, suggesting that the KChIP2 interaction site within the NTI domain may be absent in guinea pig. Without this mode of regulation present, this may explain why we do not observe a decrease in L-type Ca^2+^ current density, and instead may even be unmasking a secondary mode of regulation by KChIP2 on Cav1.2. Indeed, the KChIP2 null mice were observed to have increased Cav1.2 protein expression, despite the diminished current density [17]. This reinforces the observations here in the guinea pig where increased Cav1.2 protein expression results in a corresponding increase in *I*_*Ca*,*L*_. Notably, alternative splicing to the N-terminus of Cav1.2 can produce transcripts that omit this NTI domain [36]. While these variants predominate in endothelial cells, it does suggest KChIP2 may in fact have differential regulation on Cav1.2, depending on which variant is being expressed. That we see increased current density in the guinea pig suggests KChIP2 regulation of *I*_*Ca*,*L*_ has additional regulatory pathways yet to be determined.

The implications of cardiac KChIP2 expression grows even further when we consider its impact on *I*_*Na*_. We have previously shown that silencing KChIP2 in rat myocytes resulted in loss of *I*_*to*_ and *I*_*Na*_ [19]. Immunoprecipitation studies suggested that the subunits comprising the currents interacted to form a structural and functional subunit complex. The data we show here takes this further to say that the regulation by KChIP2 on *I*_*Na*_ occurs independently of *I*_*to*_ and can influence *I*_*Na*_ without the need to be part of a larger channel complex. This is supported by the observation that KChIP2 can coimmunoprecipitate with Nav1.5 [19]. Notably, our studies were conducted on dissociated myocytes and therefore take away the ability to study impulse propagation within native tissue, but it is intriguing to consider the influence this might have in an intact tissue.

Ultimately, our goal in this study was to characterize the changes in the cardiac action potential following KChIP2 loss, independent of any influences by *I*_*to*_. Indeed, we were able to reveal a more profound significance for KChIP2 by identifying regulation on *I*_*Ca*,*L*_ and *I*_*Na*_. These influences are especially critical in cardiac pathologies where KChIP2 loss is so common and where these currents are also modified. There are numerous electrical remodeling events in the diseased heart, and the more we understand of the multiple roles of KChIP2, the more it seems KChIP2 might be responsible in mediating those changes. Together, this establishes KChIP2 as essential in the maintenance of cardiac repolarization and depolarization, even independently from its role as a potassium channel interacting protein.

## Supporting Information

S1 Table Individual *kcnip2* expression levels across species portrayed in Fig 1. (XLSX)

S2 Table Individual APD_90_ and APD_50_ measurements at 1000 ms and 400 ms cycle lengths as portrayed in Fig 2. (XLSX)

S3 Table Individual values underlying action potential amplitude (Fig 3A), resting membrane potential (Fig 3B), and IK1 density (Fig 3D). (XLSX)

S4 Table Individual values underlying the current-voltage relationship for *I*_*Ks*_ (Fig 4A) and *I*_*Kr*_ (Fig 4B). (XLSX)

S5 Table Individual values underlying the current-voltage relationship for *I*_*Ca*,*L*_ (Fig 5B), normalized conductance measurements (Fig 5C), *I*_*Ca*,*L*_ decay kinetics (Fig 5D), and the normalized densitometry values for Cav1.2 (Fig 5E). (XLSX)

S6 Table Individual values underlying the current-voltage relationship for *I*_*Na*_ (Fig 6B), and the normalized densitometry values for Nav1.5 (Fig 6C). (XLSX)

## Abbreviations

KChIP: K+ channel interacting protein
APD: Action Potential Duration

## References

[1] An WF, Bowlby MR, Betty M, Cao J, Ling HP, Mendoza G, et al Modulation of A-type potassium channels by a family of calcium sensors. Nature. 2000;403(6769):553–6. 10676964 10.1038/35000592

[2] An RH, Wang XL, Kerem B, Benhorin J, Medina A, Goldmit M, et al Novel LQT-3 mutation affects Na+ channel activity through interactions between alpha- and beta1-subunits. Circ Res. 1998;83(2):141–6. 9686753 10.1161/01.res.83.2.141

[3] Bahring R, Dannenberg J, Peters HC, Leicher T, Pongs O, Isbrandt D. Conserved Kv4 N-terminal domain critical for effects of Kv channel- interacting protein 2.2 on channel expression and gating. The Journal of biological chemistry. 2001;276(26):23888–94. 11287421 10.1074/jbc.M101320200

[4] Decher N, Uyguner O, Scherer CR, Karaman B, Yuksel-Apak M, Busch AE, et al hKChIP2 is a functional modifier of hKv4.3 potassium channels: cloning and expression of a short hKChIP2 splice variant. Cardiovasc Res. 2001;52(2):255–64. 11684073 10.1016/s0008-6363(01)00374-1

[5] Deschenes I, DiSilvestre D, Juang GJ, Wu RC, An WF, Tomaselli GF. Regulation of Kv4.3 current by KChIP2 splice variants: a component of native cardiac I(to)? Circulation. 2002;106(4):423–9. 12135940 10.1161/01.cir.0000025417.65658.b6

[6] Patel SP, Campbell DL, Morales MJ, Strauss HC. Heterogeneous expression of KChIP2 isoforms in the ferret heart. J Physiol. 2002;539(Pt 3):649–56. 11897837 10.1113/jphysiol.2001.015156 PMC2290197

[7] Shibata R, Misonou H, Campomanes CR, Anderson AE, Schrader LA, Doliveira LC, et al A fundamental role for KChIPs in determining the molecular properties and trafficking of Kv4.2 potassium channels. The Journal of biological chemistry. 2003;278(38):36445–54. 12829703 10.1074/jbc.M306142200

[8] Morohashi Y, Hatano N, Ohya S, Takikawa R, Watabiki T, Takasugi N, et al Molecular cloning and characterization of CALP/KChIP4, a novel EF-hand protein interacting with presenilin 2 and voltage-gated potassium channel subunit Kv4. The Journal of biological chemistry. 2002;277(17):14965–75. 11847232 10.1074/jbc.M200897200

[9] Jerng HH, Pfaffinger PJ, Covarrubias M. Molecular physiology and modulation of somatodendritic A-type potassium channels. Molecular and cellular neurosciences. 2004;27(4):343–69. 15555915 10.1016/j.mcn.2004.06.011

[10] Takimoto K, Yang EK, Conforti L. Palmitoylation of KChIP splicing variants is required for efficient cell surface expression of Kv4.3 channels. The Journal of biological chemistry. 2002;277(30):26904–11. 12006572 10.1074/jbc.M203651200

[11] Pruunsild P, Timmusk T. Structure, alternative splicing, and expression of the human and mouse KCNIP gene family. Genomics. 2005;86(5):581–93. 16112838 10.1016/j.ygeno.2005.07.001

[12] Burgoyne RD. Neuronal calcium sensor proteins: generating diversity in neuronal Ca2+ signalling. Nature reviews Neuroscience. 2007;8(3):182–93. 17311005 10.1038/nrn2093 PMC1887812

[13] Buxbaum JD, Choi EK, Luo Y, Lilliehook C, Crowley AC, Merriam DE, et al Calsenilin: a calcium-binding protein that interacts with the presenilins and regulates the levels of a presenilin fragment. Nature medicine. 1998;4(10):1177–81. 9771752 10.1038/2673

[14] Carrion AM, Link WA, Ledo F, Mellstrom B, Naranjo JR. DREAM is a Ca2+-regulated transcriptional repressor. Nature. 1999;398(6722):80–4. 10078534 10.1038/18044

[15] Link WA, Ledo F, Torres B, Palczewska M, Madsen TM, Savignac M, et al Day-night changes in downstream regulatory element antagonist modulator/potassium channel interacting protein activity contribute to circadian gene expression in pineal gland. The Journal of neuroscience: the official journal of the Society for Neuroscience. 2004;24(23):5346–55. 10.1523/JNEUROSCI.1460-04.2004 PMC6729300 15190107

[16] Li H, Guo W, Mellor RL, Nerbonne JM. KChIP2 modulates the cell surface expression of Kv 1.5-encoded K(+) channels. Journal of molecular and cellular cardiology. 2005;39(1):121–32. 15878168 10.1016/j.yjmcc.2005.03.013

[17] Thomsen MB, Wang C, Ozgen N, Wang HG, Rosen MR, Pitt GS. Accessory subunit KChIP2 modulates the cardiac L-type calcium current. Circulation research. 2009;104(12):1382–9. 10.1161/CIRCRESAHA.109.196972 19461043 PMC2730599

[18] Thomsen MB, Foster E, Nguyen KH, Sosunov EA. Transcriptional and electrophysiological consequences of KChIP2-mediated regulation of CaV1.2. Channels. 2009;3(5):308–10. 19713767 10.4161/chan.3.5.9560 PMC2864922

[19] Deschenes I, Armoundas AA, Jones SP, Tomaselli GF. Post-transcriptional gene silencing of KChIP2 and Navbeta1 in neonatal rat cardiac myocytes reveals a functional association between Na and Ito currents. Journal of molecular and cellular cardiology. 2008;45(3):336–46. 10.1016/j.yjmcc.2008.05.001 18565539 PMC2580777

[20] Findlay I. Is there an A-type K+ current in guinea pig ventricular myocytes? American journal of physiology Heart and circulatory physiology. 2003;284(2):H598–604. 12388271 10.1152/ajpheart.00687.2002

[21] Ficker E, Dennis AT, Wang L, Brown AM. Role of the cytosolic chaperones Hsp70 and Hsp90 in maturation of the cardiac potassium channel HERG. Circulation research. 2003;92(12):e87–100. 12775586 10.1161/01.RES.0000079028.31393.15

[22] Rosati B, Pan Z, Lypen S, Wang HS, Cohen I, Dixon JE, et al Regulation of KChIP2 potassium channel beta subunit gene expression underlies the gradient of transient outward current in canine and human ventricle. The Journal of physiology. 2001;533(Pt 1):119–25. 11351020 10.1111/j.1469-7793.2001.0119b.x PMC2278594

[23] Teutsch C, Kondo RP, Dederko DA, Chrast J, Chien KR, Giles WR. Spatial distributions of Kv4 channels and KChip2 isoforms in the murine heart based on laser capture microdissection. Cardiovascular research. 2007;73(4):739–49. 17289005 10.1016/j.cardiores.2006.11.034

[24] Calloe K, Soltysinska E, Jespersen T, Lundby A, Antzelevitch C, Olesen SP, et al Differential effects of the transient outward K(+) current activator NS5806 in the canine left ventricle. Journal of molecular and cellular cardiology. 2010;48(1):191–200. 10.1016/j.yjmcc.2009.07.017 19632239 PMC2813348

[25] Kobayashi T, Yamada Y, Nagashima M, Seki S, Tsutsuura M, Ito Y, et al Contribution of KChIP2 to the developmental increase in transient outward current of rat cardiomyocytes. Journal of molecular and cellular cardiology. 2003;35(9):1073–82. 12967630 10.1016/s0022-2828(03)00199-8

[26] Dixon JE, Shi W, Wang HS, McDonald C, Yu H, Wymore RS, et al Role of the Kv4.3 K+ channel in ventricular muscle. A molecular correlate for the transient outward current. Circulation research. 1996;79(4):659–68. 8831489 10.1161/01.res.79.4.659

[27] Kaab S, Dixon J, Duc J, Ashen D, Nabauer M, Beuckelmann DJ, et al Molecular basis of transient outward potassium current downregulation in human heart failure: a decrease in Kv4.3 mRNA correlates with a reduction in current density. Circulation. 1998;98(14):1383–93. 9760292 10.1161/01.cir.98.14.1383

[28] Wang Y, Cheng J, Chen G, Rob F, Naseem RH, Nguyen L, et al Remodeling of outward K+ currents in pressure-overload heart failure. J Cardiovasc Electrophysiol. 2007;18(8):869–75. 17537202 10.1111/j.1540-8167.2007.00864.x

[29] Foeger NC, Wang W, Mellor RL, Nerbonne JM. Stabilization of Kv4 protein by the accessory K(+) channel interacting protein 2 (KChIP2) subunit is required for the generation of native myocardial fast transient outward K(+) currents. The Journal of physiology. 2013;591(Pt 17):4149–66. 10.1113/jphysiol.2013.255836 23713033 PMC3779109

[30] Jin H, Hadri L, Palomeque J, Morel C, Karakikes I, Kaprielian R, et al KChIP2 attenuates cardiac hypertrophy through regulation of Ito and intracellular calcium signaling. Journal of molecular and cellular cardiology. 2010;48(6):1169–79. 10.1016/j.yjmcc.2009.12.019 20051248 PMC2866822

[31] Sah R, Ramirez RJ, Oudit GY, Gidrewicz D, Trivieri MG, Zobel C, et al Regulation of cardiac excitation-contraction coupling by action potential repolarization: role of the transient outward potassium current (I(to)). The Journal of physiology. 2003;546(Pt 1):5–18. 12509475 10.1113/jphysiol.2002.026468 PMC2342473

[32] Greenstein JL, Wu R, Po S, Tomaselli GF, Winslow RL. Role of the calcium-independent transient outward current I(to1) in shaping action potential morphology and duration. Circulation research. 2000;87(11):1026–33. 11090548 10.1161/01.res.87.11.1026

[33] Aiba T, Tomaselli GF. Electrical remodeling in the failing heart. Current opinion in cardiology. 2010;25(1):29–36. 10.1097/HCO.0b013e328333d3d6 19907317 PMC2855498

[34] Cordeiro JM, Calloe K, Moise NS, Kornreich B, Giannandrea D, Di Diego JM, et al Physiological consequences of transient outward K+ current activation during heart failure in the canine left ventricle. Journal of molecular and cellular cardiology. 2012;52(6):1291–8. 10.1016/j.yjmcc.2012.03.001 22434032 PMC3401930

[35] Hill JA, Olson EN. Cardiac plasticity. The New England journal of medicine. 2008;358(13):1370–80. 10.1056/NEJMra072139 18367740

[36] Kanevsky N, Dascal N. Regulation of maximal open probability is a separable function of Ca(v)beta subunit in L-type Ca2+ channel, dependent on NH2 terminus of alpha1C (Ca(v)1.2alpha). The Journal of general physiology. 2006;128(1):15–36. 16801381 10.1085/jgp.200609485 PMC2151559

